# Tuberculosis Dactylitis in a Patient With Rheumatoid Arthritis: A Case Report

**DOI:** 10.7759/cureus.74569

**Published:** 2024-11-27

**Authors:** Andre Pinto, Joaquim Pereira, Francisco Caneira, Duarte Garcao, Bruno Rosa

**Affiliations:** 1 Plastic and Reconstructive Surgery, Hospital de Santa Maria, Unidade Local de Saúde Santa Maria (ULSSM), Lisbon, PRT; 2 Rheumatology, Hospital de Santa Maria, Unidade Local de Saúde Santa Maria (ULSSM), Lisbon, PRT

**Keywords:** extrapulmonary tuberculosis (eptb), hand tumour, plastic hand surgery, rheumatoid arthriitis, tuberculosis dactylitis

## Abstract

Tuberculosis (TB) dactylitis of the hand is a rare and challenging pathology, requiring positive bacterial identification through culture or biopsy for diagnosis. Treatment is also challenging, although it typically yields an excellent response to long-term tuberculostatic therapy. We describe a case of osteoarticular tuberculous dactylitis in a 36-year-old woman with rheumatoid arthritis (RA) and a history of lymphoma. The suspicion arose from an insidious, painless swelling of the proximal interphalangeal joint of the second digit of the right hand in a patient undergoing methotrexate treatment for RA. Under a long-term anti-TB multi-drug regimen, the patient’s signs and symptoms progressively improved, and hand function was nearly fully restored. The authors discuss the diagnostic and surgical treatment challenges encountered in plastic and reconstructive surgery care.

## Introduction

Osteoarticular tuberculosis (TB) is an extrapulmonary TB manifestation, representing approximately 5% of TB cases [1]. The vast majority of patients present with either vertebral spine or large joint involvement. TB dactylitis is an exceptional presentation of this disease [1,2]. The small size of the bones of the hand might be a major factor explaining the rarity of such a presentation [3]. Insidious swelling and minor or no pain are frequent and contribute to a delayed diagnosis. Patients with rheumatoid arthritis (RA) who are undergoing immunosuppression are at risk of developing opportunistic osteoarticular infections, which may be mistaken for disease activity in the initial stages. HIV infection, chronic liver disease, chronic kidney disfunction, malnutrition, diabetes mellitus, alcohol, tobacco and steroid therapy are additional known risk factors for extrapulmonary TB [4-6].

## Case presentation

**Figure 1:**
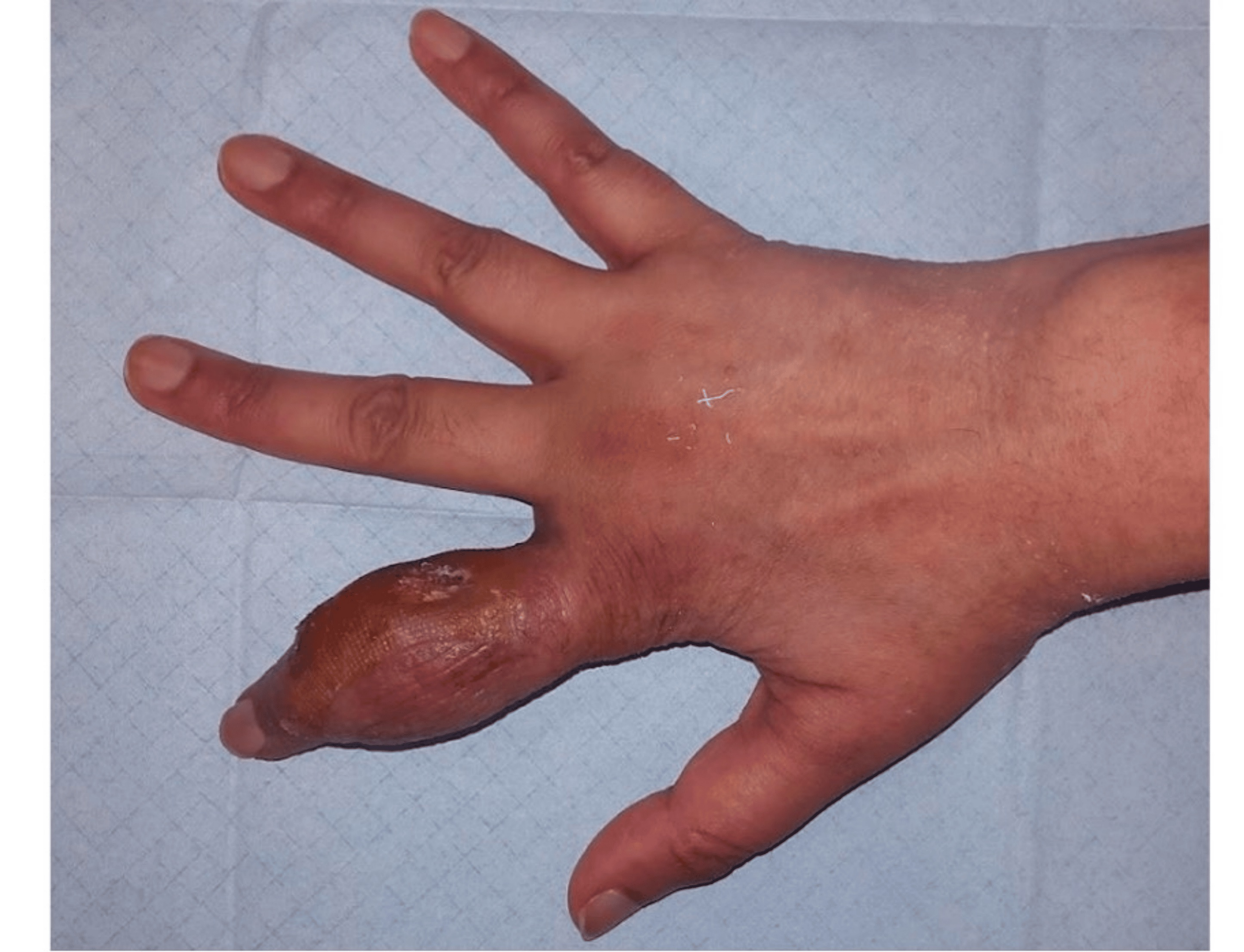
Initial presentation. Tumor of the proximal interphalangeal joint of the second finger of the right hand with inflammation signs.

A 35-year-old woman of Indian nationality, with a history of RA, nodular sclerosing Hodgkin lymphoma stage IVB in current remission, and chemotherapy-induced restrictive lung disease, presented to our center with chronic polyarthritis involving the bilateral proximal interphalangeal (PIP) joints of the hands, wrists, shoulders, and hips. The seropositive RA diagnosis was established three years before, and she was treated with prednisolone 2.5 mg per day and methotrexate (MTX) 15 mg per week. After more than two years in remission under treatment with MTX, she developed progressive swelling, redness, and warmth of the PIP joint of the second finger of her right hand, as illustrated in Figure 1.

**Figure 2:**
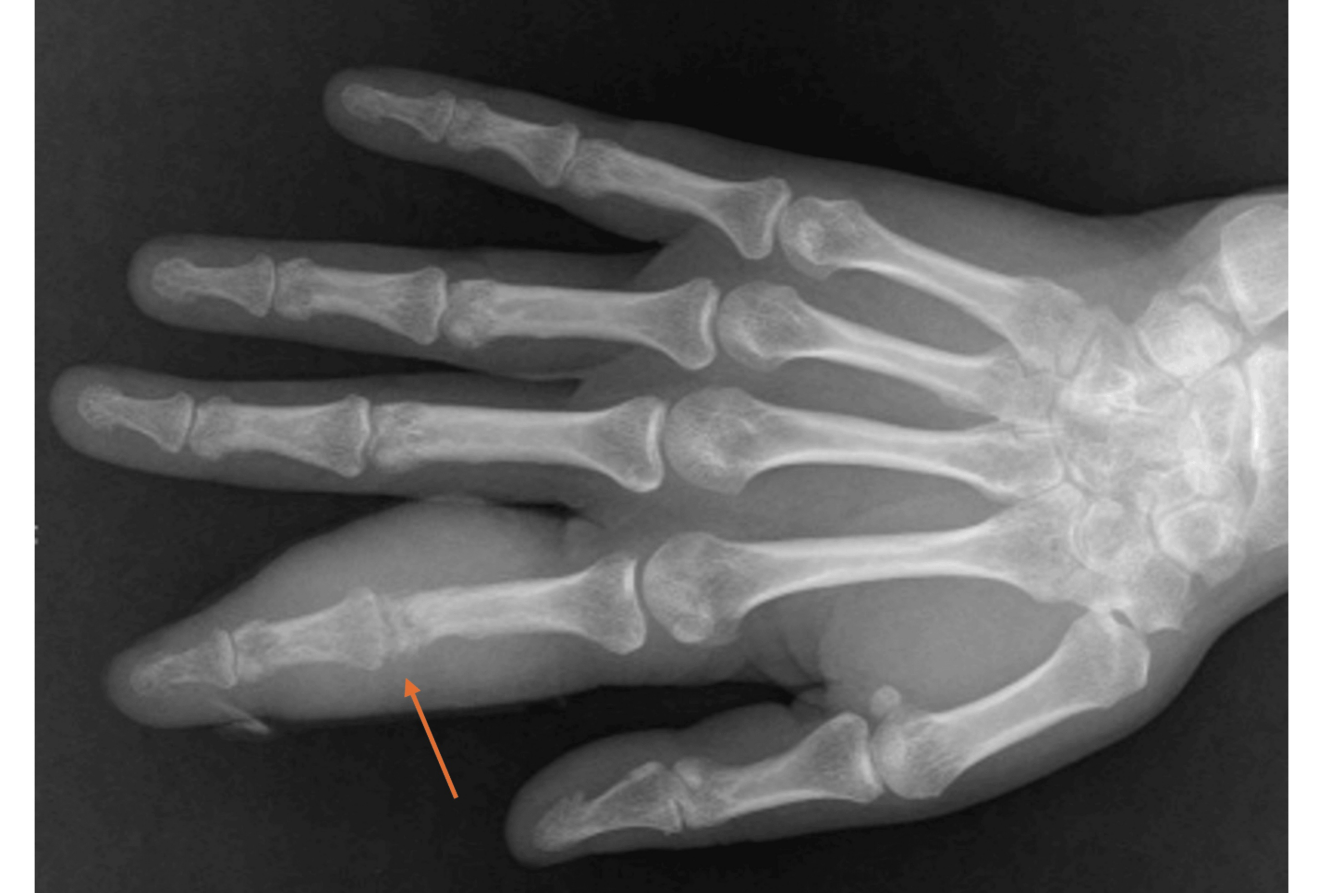
X-ray of the right hand. The arrow pointing to the tumor-associated erosion of the proximal interphalangeal joint of the second finger.

Both erythrocyte sedimentation rate (ESR) and C-reactive protein (CRP) were normal. The Quantiferon-TB test was positive. A roentgenogram showed destruction of the PIP joint with potential osteitis and opacification of soft tissues, as shown in Figure 2. Musculoskeletal ultrasound revealed extensive synovitis of the second right-hand PIP joint, significant finger flexor tenosynovitis, a grade 3 Doppler signal, and contiguous bone irregularities of the intermediate phalanx.

Simultaneously, she presented a supraclavicular adenopathy. Given the suspicion of lymphoma relapse, an excisional biopsy of the adenopathy was performed, revealing granulomas with caseating necrosis. She was referred to the Plastic Surgery department for eventual surgical management, including both drainage and biopsy, with a high suspicion of osteoarticular TB.

The patient presented with swelling, redness, and warmth in the PIP joint of the second finger of her right hand. There was pain under palpation or flexion of the joint, limiting the range of motion. The swollen tissue was soft on touch, and a subcutaneous collection was apparent under a domal apex present on the lesion.

Under local anesthesia, a domal surgical drainage of the swollen tissue was attempted, but it was unsuccessful because the mass appeared proliferative in nature, and there was no significant joint effusion.

**Figure 3:**
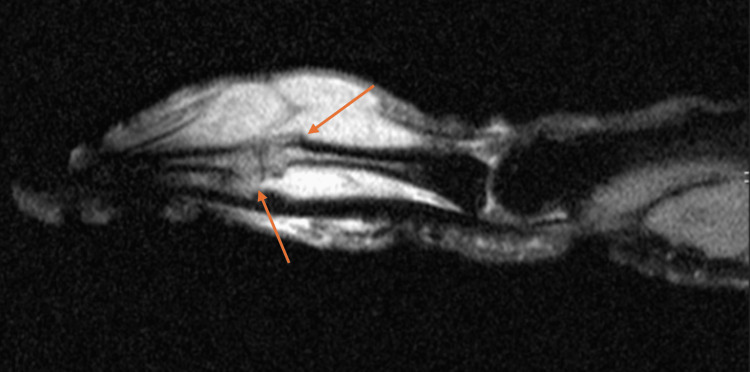
Lateral MRI image of the second finger. Arrows pointing to synovitis and bone injury on the proximal and middle phalanxes.

**Figure 4:**
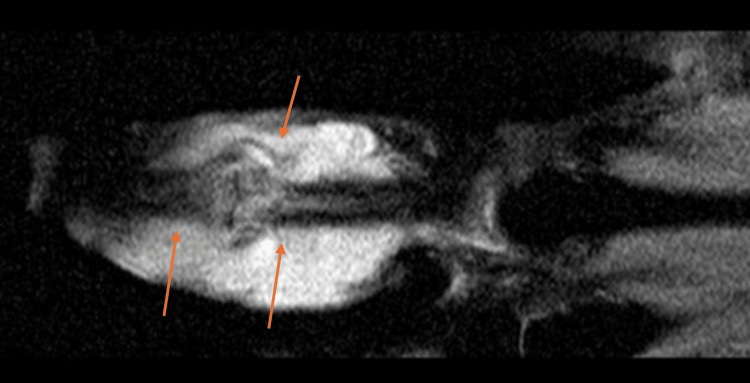
Anteroposterior view of the second finger. Arrows pointing to synovitis and bone injury on the proximal and middle phalanxes.

An MRI study showed extensive synovitis with sparse cystic areas and bony extension of the disease affecting both the proximal and intermediate phalanges of the second finger (Figures 3-4).

Under local anesthesia, the patient underwent an incisional biopsy, and samples were collected for both microbiological and pathological analysis.

The pathology study revealed caseating granulomatous necrosis, and anti-TB medical treatment was initiated. Later, Mycobacterium tuberculosis grew on cultures, confirming the diagnosis of osteoarticular TB.

She was treated with tuberculostatics for one year, after which there was complete resolution of the digital swelling.

## Discussion

In this case, the overlapping clinical findings of osteoarticular TB and RA increased the likelihood of misdiagnosis and potentially led to differing appropriate care. The nature of the diagnosis, being a very atypical manifestation of TB, especially as a monoarticular form, made it a difficult case to diagnose [7,8].

A progressively worsening chronic monoarthritis with warmth alerted the clinicians to an alternate cause of arthritis in a patient with RA. Patients with RA under immunosuppression, particularly those on TNF inhibitors, are at increased risk of osteoarticular TB. Also, the discovery of caseating necrosis and granulomas in the adenopathy biopsy, along with a positive Quantiferon-TB test, highlighted the likely TB nature of the disease. However, the complete establishment of the diagnosis requires positive laboratory cultures, which take several weeks [3,6]. Under these circumstances, we opted to initiate the anti-TB treatment.

**Figure 5:**
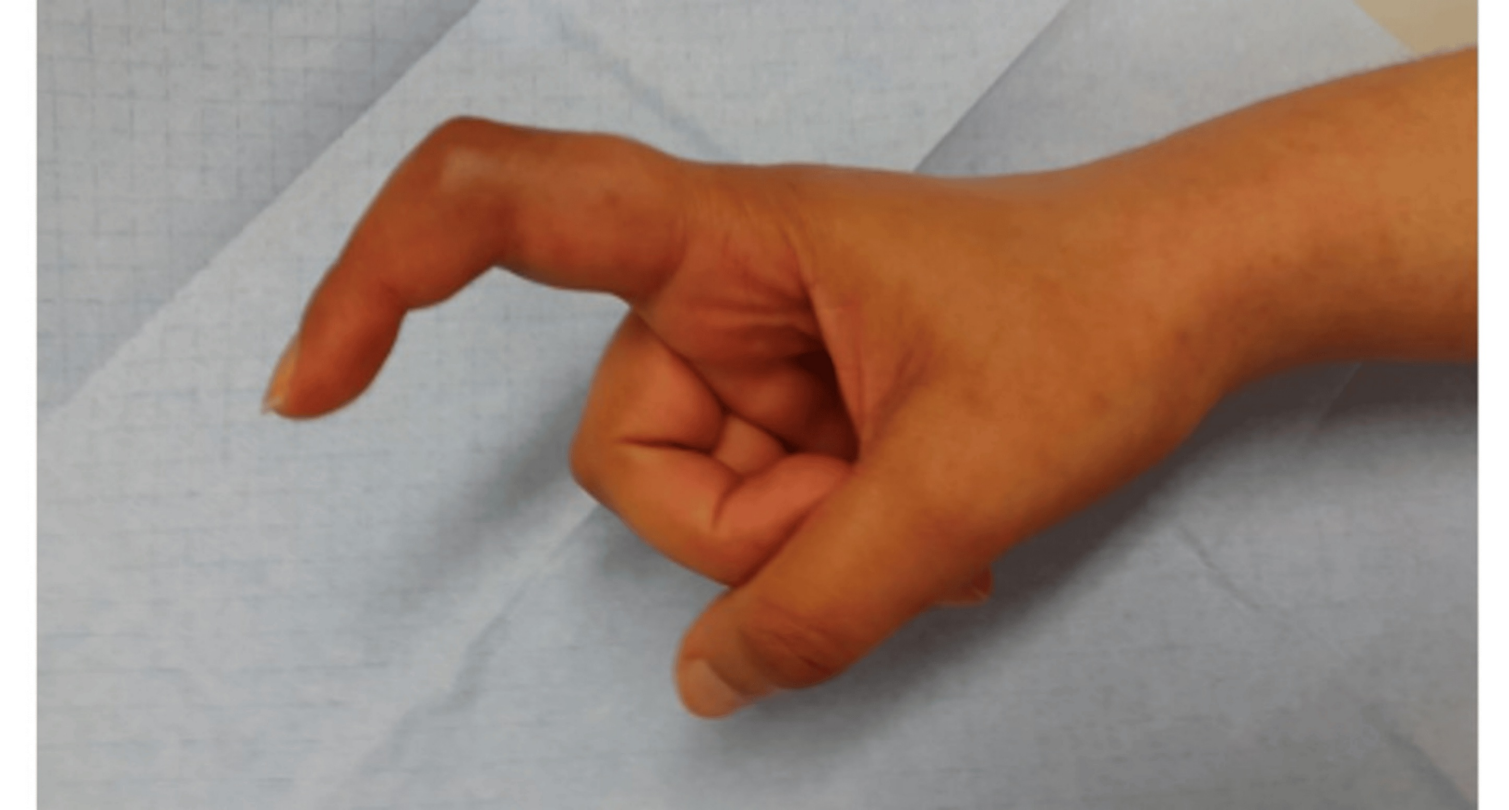
Arthrosis of the proximal interphalangeal joint one year after completing the course of treatment.

As a result, one year after completing the course of treatment, the patient developed a fibrotic 30-degree flexion contracture of the PIP joint, causing minor functional impairment. However, she tolerated it without significant limitations in daily activities, as shown in Figure 5.

## Conclusions

TB dactylitis is an atypical manifestation of TB, especially when it presents as a monoarticular case. A definitive TB dactylitis diagnosis implies positive cultures, depending on a high index of suspicion. Particularly in endemic populations, appropriate detection to enable early treatment is essential.

## References

[1] Latief W, Asril E (2019). Tuberculosis of the wrist mimicking rheumatoid arthritis - a rare case. Int J Surg Case Rep.

[2] Basnayake O, Nihaj A, Pitagampalage R, Mendis H (2019). Tuberculosis presenting as isolated wrist swelling: a case report and review of literature. Case Rep Surg.

[3] Fairag R, Hamdi A (2016). Tuberculous dactylitis: case presentation and functional outcome. J Orthop Case Rep.

[4] Lee JY (2015). Diagnosis and treatment of extrapulmonary tuberculosis. Tuberc Respir Dis (Seoul).

[5] Hunfeld KP, Rittmeister M, Wichelhaus TA, Brade V, Enzensberger R (1998). Two cases of chronic arthritis of the forearm due to Mycobacterium tuberculosis. Eur J Clin Microbiol Infect Dis.

[6] Abebe W, Abebe B, Molla K, Alemayehu T (2016). Tuberculous dactylitis: an uncommon presentation of skeletal tuberculosis. Ethiop J Health Sci.

[7] Wang T, Zhao G, Rui YJ, Mi JY (2018). Successfully treating hand primary tuberculous synovitis by synovectomy combined antituberculous therapy: a case report. Medicine (Baltimore).

[8] Pigrau-Serrallach C, Rodríguez-Pardo D (2013). Bone and joint tuberculosis. Eur Spine J.

